# Composition and Activity of Microbial Communities along the Redox Gradient of an Alkaline, Hypersaline, Lake

**DOI:** 10.3389/fmicb.2018.00014

**Published:** 2018-01-31

**Authors:** Christian F. Edwardson, James T. Hollibaugh

**Affiliations:** ^1^Department of Marine Sciences, University of Georgia, Athens, GA, United States; ^2^Department of Microbiology, University of Georgia, Athens, GA, United States

**Keywords:** Mono Lake, soda lakes, alkaliphile, halophile, Picocystis

## Abstract

We compared the composition of microbial communities obtained by sequencing 16S rRNA gene amplicons with taxonomy derived from metatranscriptomes from the same samples. Samples were collected from alkaline, hypersaline Mono Lake, California, USA at five depths that captured the major redox zones of the lake during the onset of meromixis. The prokaryotic community was dominated by bacteria from the phyla Proteobacteria, Firmicutes, and Bacteroidetes, while the picoeukaryotic chlorophyte *Picocystis* dominated the eukaryotes. Most (80%) of the abundant (>1% relative abundance) OTUs recovered as amplicons of 16S rRNA genes have been reported in previous surveys, indicating that Mono Lake's microbial community has remained stable over 12 years that have included periods of regular, annual overturn interspersed by episodes of prolonged meromixis that result in extremely reducing conditions in bottom water. Metatranscriptomic sequences binned predominately to the Gammaproteobacteria genera *Thioalkalivibrio* (4–13%) and *Thioalkalimicrobium* (0–14%); and to the Firmicutes genera *Dethiobacter* (0–5%) and *Clostridium* (1–4%), which were also abundant in the 16S rRNA gene amplicon libraries. This study provides insight into the taxonomic affiliations of transcriptionally active communities of the lake's water column under different redox conditions.

## Introduction

Mono Lake, located in California, USA east of the Sierra Nevada Mountains on the western edge of the Great Basin, has been studied extensively due to its unusual chemistry and biology (reviewed in Melack et al., 2017). Mono Lake is an athalassic hypersaline (~90 g/L) and alkaline (pH ~9.8) terminal lake with geothermal inputs leading to elevated arsenic concentrations (~200 μM). Thermal stratification leads to the formation of a seasonal oxycline and to anoxic conditions in the lake's hypolimnion. The lake is generally monomictic, but undergoes periods of prolonged stratification (meromixis) following wet winters (Melack and Jellison, 1998). Microbial respiration below the oxycline leads to the reduction of arsenate to arsenite. Sulfide accumulation, especially in the monimolimnion, leads to the formation of thioarsenic compounds (Hollibaugh et al., 2005).

Several studies of the lake's biogeochemistry have focused on the role of microorganisms in arsenic geochemistry (reviewed in Oremland et al., 2004, 2017). These studies have used isolates (Switzer-Blum et al., 1998; Sorokin et al., 2002; Hoeft et al., 2004, 2007; Fisher and Hollibaugh, 2008), enrichment cultures (Hollibaugh et al., 2006; Budinoff and Hollibaugh, 2008; Fisher and Hollibaugh, 2008; Edwardson et al., 2014), and clone libraries of functional gene amplicons (Giri et al., 2004; Lin et al., 2005; Nercessian et al., 2005; Scholten et al., 2005; Lecleir et al., 2007). A more recent study (Edwardson and Hollibaugh, 2017) used metatranscriptomics to analyze microbial communities active in the As and S biogeochemical cycles in the lake. Other aspects of the biogeochemistry of Mono Lake that have been studied include ammonia and methane oxidation (Joye et al., 1999; Ward et al., 2000; Lin et al., 2005), sulfur cycling (Scholten et al., 2005; Hollibaugh et al., 2006), chitin degradation (Lecleir et al., 2004), and carbon fixation (Giri et al., 2004; Oremland et al., 2017).

The composition, diversity and distribution of microbes in aquatic communities are important ecological parameters, reflecting both functional aspects of these assemblages as well as the influence of environmental conditions on the survival and growth of specific organisms (Fuhrman et al., 2015). Analysis of 16S rRNA gene sequences has emerged as the method of choice for studying the composition of microbial communities (e.g., Fierer and Jackson, 2006; Sogin et al., 2006; Gilbert et al., 2012). Community composition has been studied in extreme environments, including Mono Lake, using libraries of cloned 16S rRNA gene amplicons (Humayoun et al., 2003; Foti et al., 2008; Mesbah et al., 2008; Antony et al., 2014), despite limited sequencing depth afforded by the necessity of cloning amplicons. The deeper coverage of amplicon libraries afforded by high-throughput sequencing provides a more detailed and complete analysis of microbial community composition and dynamics (Harris et al., 2013; Lanzén et al., 2013; Schneider et al., 2013; Yelton et al., 2013; Podell et al., 2014; Rascovan et al., 2016). Metagenomic surveys (e.g., Venter et al., 2004; Rusch et al., 2007) provide an alternative approach for assessing the abundance and taxonomic composition of microbial communities, while surveys of genes transcribed (mRNA) by microbial communities (metatranscriptomics, e.g., Poretsky et al., 2006; Gifford et al., 2011; Satinsky et al., 2014) allows identification of both the organisms and pathways that may be biogeochemically active in a given community.

Previous studies of the taxonomic composition (Humayoun et al., 2003) and environmental metatranscriptomics (Poretsky et al., 2005) of Mono Lake microbial communities were based on analysis of cloned amplicons sequenced by the dideoxy chain termination technique (Sanger et al., 1977), yielding limited sequencing depth. We were interested in comparing the composition of microbial communities in the lake at the onset of stratification in 2012 with an assessment of community composition made at the end of a 5-year period of meromixis (Humayoun et al., 2003). We were also interested in comparing the distribution of transcriptionally active microorganisms (Edwardson and Hollibaugh, 2017) along the lake's redox gradient with distributions of their genomes as reflected by the distribution of 16S rRNA genes in deeply sequenced libraries of PCR amplicons, and to evaluate changes in the apparent activity of specific organisms over the range of redox conditions found in the Mono Lake water column.

## Materials and methods

### Field site and sampling

**Figure 1:**
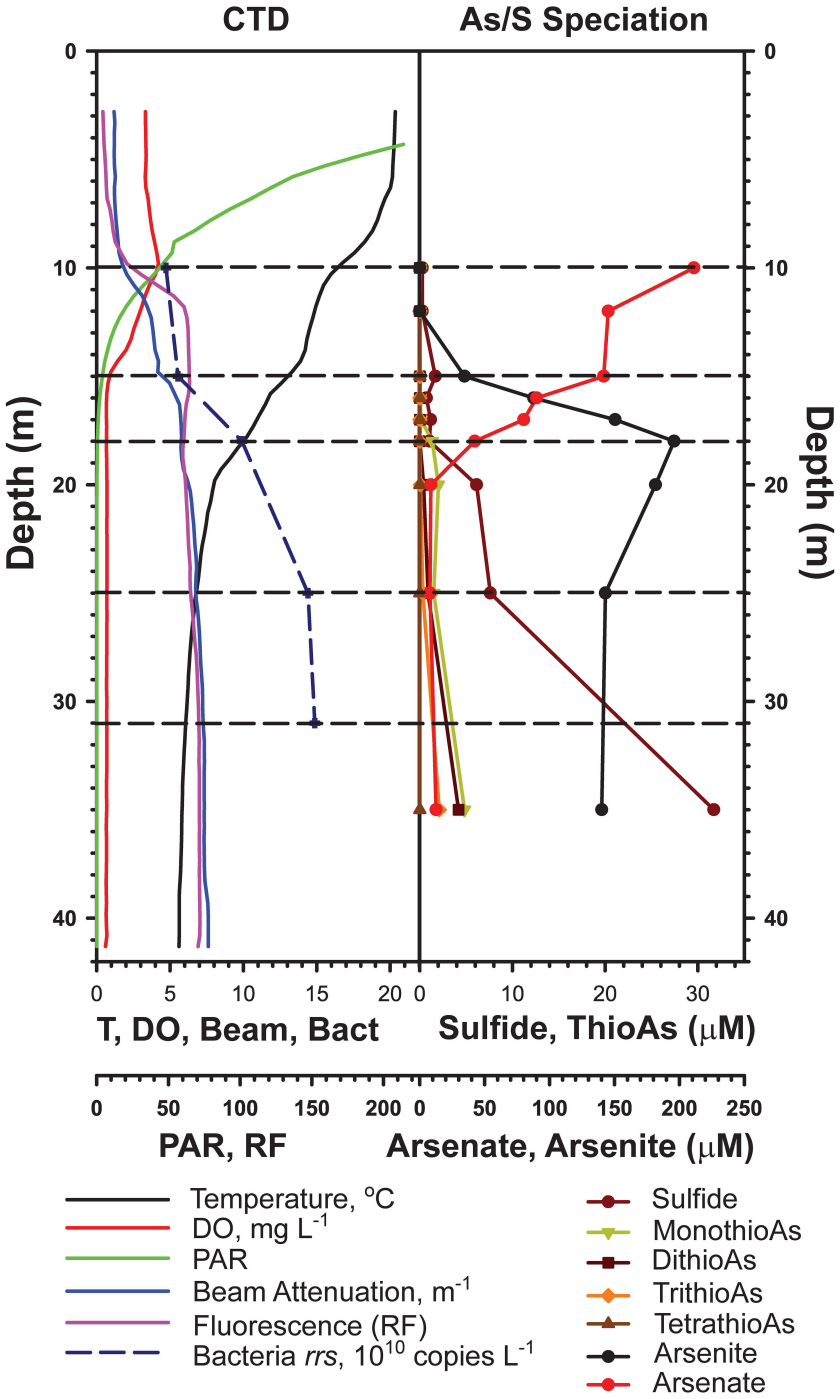
Water column profiles of physicochemical variables measured on July 12, 2012 (from Edwardson and Hollibaugh, 2017).

The samples used in this study were collected during summer (July 12 and 13, 2012, lake surface elevation: 1945 m) following a winter when the lake did not mix fully (the onset of meromixis). We sampled at Station 6 (37.964167° N,−119.022000° W), in the deepest basin of the lake, in order to capture the full redox gradient and to minimize the influence of breaking internal waves on vertical mixing (Vidal et al., 2013) and hence the distribution and activity of microbes. Duplicate samples were collected from depths of 10, 15, 18, 25, and 31 m to sample communities from the major redox zones of the lake (see Figure 1). Samples were taken as described previously (Hollibaugh et al., 2005; Edwardson and Hollibaugh, 2017). Briefly, vertical profiles of relevant environmental variables: photosynthetically active radiation (PAR, LiCor 2π quantum sensor, 400–700 nm, μE m^−2^ s^−2^), fluorescence (WetLabs fluorometer, relative fluorescence units), dissolved oxygen (SBE 43, mg L^−1^) and attenuation coefficient (WetLabs transmissometer, 600 nm wavelength light source, 10 cm path length, m^−1^) were obtained using sensors mounted on the frame holding a SeaBird SBE 19 CTD (conductivity, temperature, depth) recorder, modified and calibrated for use in Mono Lake. Water samples for total sulfide analysis (Cline, 1969) and arsenic speciation (Planer-Friedrich et al., 2007) were collected as described previously (Hollibaugh et al., 2005; Edwardson and Hollibaugh, 2017).

### Nucleic acid sampling and processing

Water for 16S rRNA gene analysis and synthesis of subtractive hybridization probes (see below) was collected from 5 L Niskin bottles into foil-wrapped polycarbonate bottles and stored in an insulated cooler on ice until processed further. Septum-capped bottles were filled to leave no head space and only one sample was taken from each depth. Water was filtered through Sterivex GV, 0.22 μm pore-size, cartridge filters (EMD Millipore, Billerica, MA) using a peristaltic pump within 8 h of collection. DNA was extracted from the filters using a lysozyme, proteinase K, sodium dodecyl sulfate digestion, followed by phenol-chloroform extraction as described previously (Kalanetra et al., 2009). Quantitative PCR (qPCR) of Bacteria 16S rRNA genes in this DNA was used to estimate the depth distribution of prokaryotes in the lake at the time of sampling (Kalanetra et al., 2009).

Samples for mRNA analysis were collected by pumping water from 31 m on July 12 and from 10 to 25 m on July 13. Duplicate samples of total particulate RNA (~0.5–2 L) were pumped sequentially from the sampling depth through 142 mm diameter, 0.2 μm pore-size Supor membrane filters. The filters were placed in 15 mL polypropylene centrifuge tubes and immediately frozen in liquid nitrogen, then transported to the laboratory where they were stored at −80°C until extracted. The elapsed time from beginning the filtration at a given depth until the filter was placed in liquid nitrogen was <15 min. Total RNA was extracted from particles retained by the filters using bead beating and RNEasy Mini Kits (Gifford et al., 2011; Edwardson and Hollibaugh, 2017), with internal standards added as described previously (Satinsky et al., 2013).

### 16S rRNA gene sequencing

We analyzed the distribution of 16S rRNA genes in each of our samples by sequencing libraries of PCR amplicons using 454 Pyrosequencing (Roche Diagnostics) technology (“pyrosequences” or “pyrosequenced libraries” hereinafter). We amplified the V4-V5 region of the 16S rRNA gene using primers 515F (Caporaso et al., 2011) and 907R (Armitage et al., 2012) that amplify both Bacteria and Archaea 16S rRNA genes. The forward primer also contained the Roche LIB-L Adapter A and a 10 nt MID tag on the 5′ end. A different MID tag was used for each depth (two samples per MID tag). The reverse primer contained the Roche LIB-L Adapter B on the 5′ end. PCR was performed in triplicate reactions for each sample using Q5® High-Fidelity polymerase (NEB). Each 25 μL reaction contained the following: Q5 Reaction Buffer (5 μL of 5X), 200 nM of each primer, 200 μM each dNTP and 0.02 U/μL of Q5 polymerase, and 1 μL (12–61 ng) DNA template. The PCR program used was: 98°C for 30 s, 25 cycles of 98°C for 10 s, 60°C for 10 s and 72°C for 10 s, with a final step of 72°C for 2 min. Individual PCR samples were run on a 1% agarose gel and single bands of the expected length were cut and extracted using a QiaQuick Spin kit (Qiagen). The triplicates were pooled and an additional cleanup step was performed with a QiaQuick Spin kit to concentrate them. A final cleanup was performed using the AmpureXP kit (Beckman Genomics). Products were quantified using the PicoGreen kit (Life Technologies) and pooled at an equivalent weight. Samples were sequenced on approximately 1/60th of a plate at the Georgia Genomics Facility using 454 Titanium® chemistry.

### Metatranscriptomes

Ribosomal RNA was depleted from the RNA pool using subtractive hybridization probes (Stewart et al., 2010) synthesized from Mono Lake DNA collected as described above. Probes were synthesized from PCR products amplified using the primers described in Stewart et al. (2010), but with 25 μL reactions using Q5® High-Fidelity polymerase (NEB) following the manufacturers recommended reaction conditions and a modified amplification protocol: 98°C for 30 s, 30 cycles of 98°C for 10 s, annealing (Eub16S: 63°C, Eub23S: 55°C, Arch16S: 67°C, Arch23S: 64°C, Euk18S: 65°C, Euk28S: 61°C) for 30 s, 72°C for 40, 60, or 80 s (16/18S, 23S, and 28S reactions, respectively), and 72°C for 2 min. One PCR reaction was performed for each DNA replicate from each depth (*n* = 10) and all were pooled and purified with a QiaQuick PCR Cleanup kit (Qiagen) and concentrated using a SpeedVac 120 (Savant), then a second round of purification was performed with the AmpureXP kit (Beckman Genomics) to remove remaining primers and primer dimers. The Arch16S PCR did not yield a usable product and thus was excluded from probe synthesis. Ribosomal RNA-depleted samples were amplified using the MessageAmpII-Bacteria kit (Ambion). Double stranded cDNA was prepared using a combination of first and second strand kits: SuperScript III First Strand Synthesis (Life Technologies) primed with random hexamers, and NEBNext mRNA Second Strand synthesis module (NEB). Double stranded cDNA was purified with a PureLink PCR cleanup kit (Life Technologies) followed by ethanol precipitation. The cDNA was sheared (Covaris instrument) to a targeted ~225 bp insert size, and libraries were prepared using the TruSeq DNA (Illumina) kit with custom indices developed by the Georgia Genomics Facility at the University of Georgia. Samples were pooled and sequenced on one lane of an Illumina HiSeq2500 in Rapid Run mode with the 150PE (300 cycle) kit at HudsonAlpha Genomic Services Lab (Huntsville, AL).

### Bioinformatics of 16S rRNA sequences

Sequences were processed using QIIME versions 1.5 (initial sample processing and de-noising) and 1.8 (OTU picking and taxonomic assignment) (Caporaso et al., 2010). Samples were split and filtered using default quality control settings, with the additional removal of all reads with ambiguous bases (Huse et al., 2007) and all reads longer than 500 bp. Reads were de-noised using Denoiser (Reeder and Knight, 2010) and checked for chimeras using USEARCH 6.1 *de novo* chimera picking (UCHIME) (Edgar, 2010; Edgar et al., 2011). OTUs were picked using the open reference method with USEARCH 6.1 at a 97% identity cutoff. The SILVA rRNA database, release 111 (Quast et al., 2013), was used for reference-based OTU picking and for taxonomic assignment using UCLUST (Edgar, 2010). Additional taxonomic assignments for representative sequences of OTUs that remained “unassigned” after this step were made using SINA (Pruesse et al., 2012) against SILVA release 119, or using the RDP classifier (Wang et al., 2007). The QIIME taxonomic assignments of a number of sequences were compared to SINA taxonomic assignments to verify of their QIIME assignments. Chloroplast, mitochondria, and singleton OTUs, as well as OTUs that failed to align with the SILVA 16S reference alignment were removed. Representative sequences of each OTU were used to search against the NCBI nr database using BLASTN (Altschul et al., 1990).

Sequences analyzed in a previous study of the microbial diversity of Mono Lake by Humayoun et al. (2003), who sampled different depths from the same redox zones at Station 6 (2 m, aerobic; 17.5 m, microaerophilic, 23 m, anoxic; and 35 m, sulfidic, see Humayoun et al., 2003), were downloaded from NCBI GenBank (*n* = 274). All sequences that spanned the region amplified by the 16S rRNA gene primers we used here (515F/907R) were aligned in Geneious and trimmed to the length of the pyrosequenced amplicons (~375 nt, *n* = 174). These sequences were processed in QIIME as described above to determine OTUs at 97% identity and to define the taxonomy of sequences from the Humayoun et al. (2003) study in the same manner as our pyrosequences. We retained singletons (*n* = 48) in this analysis due to the small size of the Humayoun et al. (2003) database. The number of sequences assigned to each OTU was determined manually. We also included sequences from other stations and depths sampled during that same study that were available in GenBank but not analyzed by Humayoun et al. (2003). Relative abundances of each OTU were determined for each depth at Station 6. Alpha and beta diversity analyses were performed on the 454 amplicons using the R package *phyloseq* (McMurdie and Holmes, 2013). Reads were randomly subsampled to the lowest number of reads per sample (1,588) prior to alpha and beta diversity analysis. Raw reads were deposited in the NCBI SRA under accession number SRP074130 (BioProject PRJNA319794).

### Bioinformatics analysis of metatranscriptomes

FASTQ files were paired using PEAR (version 0.9.2; Zhang et al., 2014) with a minimum overlap of 1 and no statistical tests. PRINSEQ (version 0.20.3; Schmieder and Edwards, 2011) was used to trim and perform quality control using the following parameters: 10–90% GC content, minimum length 35 bp, mean quality score of 20, trim from 3′ to 5′ ends with a sliding scale window of 3 and step of 1 with a minimum mean quality score of 20, trim >5 uncalled bases from ends, trim >5 A/T tails, and allow only 1 uncalled base (any reads with uncalled bases were later removed). RiboPicker (version 0.4.3; Schmieder et al., 2012) was used to identify rRNA reads in metatranscriptome libraries. The default parameters (50% coverage, 75% identity, 30 base align length, BWA-SW Z-best value of 3) and the non-redundant rRNA database were used. FASTQ files were converted to FASTA files using FASTX-Toolkit (http://hannonlab.cshl.edu/fastx_toolkit/) default settings, which removes any reads that contain uncalled bases.

A custom BLAST database containing sequences of internal standards provided by Satinsky and Moran (Satinsky et al., 2014) was created using BLAST+ (Camacho et al., 2009). A BLASTN (Altschul et al., 1990) search against the custom database was used to count reads assigned to internal standards. Recovery was calculated using counts of hits to sequences of standards with bit scores >50, divided by the number of internal standard sequences added to each sample (Satinsky et al., 2013). These sequences were then removed from the input FASTA files using a QIIME script (filter_fasta.py; Caporaso et al., 2010). A local RapSearch2 (Zhao et al., 2012) database of all protein sequences in the RefSeq (Tatusova et al., 2014) database (release 64) was downloaded from NCBI. Rapsearch2 was used to annotate metatranscriptome reads using an *e*-value cutoff of 10^−5^, keeping only the top hit. Further processing removed all hits with bit scores <40 (Gifford et al., 2011). Custom scripts were used to fully annotate and assign taxonomy to hits. Absolute abundances (transcripts/L) of transcripts for each unique gene (locus_tag) were calculated by multiplying count of hits to that locus_tag in each library by the factor determined from recovery of internal standard reads, divided by volume filtered for each sample. Relative abundance of each unique transcript (locus_tag) in each library (% of all transcripts in that library) was calculated as the absolute abundance of that transcript divided by the total number of transcripts in that library. We used averages of these relative abundances for the 2 libraries from each depth in subsequent comparisons.

We performed an additional screening step to focus our analysis confidently on protein-coding transcripts. Hits to records with RefSeq annotations that contained the terms “hypothetical” or “putative” were removed from the transcript data set manually using text searches. This filter likely removed hits to some transcripts that encode valid proteins whose function is either not known or that are annotated incorrectly, but it also removed incorrect assignments to non-protein encoding transcripts (Tripp et al., 2011). Disproportionally frequent hits to “cell wall hydrolases” were also found in the dataset. In one case (library 31A), the top hit to this annotation accounted for 11% of all Bacteria transcripts, whereas the largest bin for sequences that did not contain this term only accounted for 2% of the transcripts in the library. We analyzed each of the hits that made up >1% of “hypothetical/cell wall hydrolases” further using a TBLASTN search against the nr/nt nucleotide database to determine the identity of the best hit to the RefSeq protein database. This analysis (not shown) revealed misannotated ribosomal RNAs, small RNAs or ribozymes, as has been reported previously (Tripp et al., 2011). Thus, annotations containing the following words or phrases: “hydrolase,” “predicted protein,” “uncharacterized protein,” and “cell wall-associated hydrolase” were deemed to be inaccurate and hits to them were removed from the data set. Reads are deposited in the NCBI SRA under accession number SRP068308 (BioProject PRJNA308451).

### Phylogenetic analysis

The phylogeny of the 16S rRNA gene sequences obtained from pyrosequencing was compared with phylogeny from the sequences obtained by Sanger-sequencing cloned amplicons from Humayoun et al. (2003) as follows. Representative sequences from all OTUs that accounted for >1% relative abundance in the pyrosequence database (*n* = 34) were aligned with OTUs with >1% relative abundance in the Sanger sequence database (*n* = 60), and with 16S rRNA genes retrieved from genomes corresponding to taxa that represented >1% relative abundance in the metatranscriptomic dataset. All sequences were trimmed to the length of the pyrosequenced reads (~375 bp) and aligned using the SINA aligner (Pruesse et al., 2012). Alignments were imported into Geneious 8 (Kearse et al., 2012), and neighbor-joining consensus trees (Jukes-Cantor distances) with 100 bootstrap replicates were built, with *Halobacterium salinarum* as the outgroup. Three separate trees (Proteobacteria, Firmicutes, and Other Phyla) were constructed in this manner.

## Results

### Chemical characteristics of mono lake

Mono Lake Station 6, the site of many previous microbiological studies of the lake, was sampled at five discrete depths chosen based on the chemical profile of the lake at the time of sampling (Edwardson and Hollibaugh, 2017, Figure 1). The epilimnion is characterized by the highest temperatures (>15°C), highest light, highest dissolved oxygen concentrations, and is subject to intense grazing by brine shrimp, *Artemia monica* (Jellison and Melack, 1993). We sampled at the base of the epilimnion (10 m), the base of the oxycline (15 m), and near the base of the thermocline (18 m). The dissolved oxygen concentration was 0.83 mg L^−1^ at 15 m, and decreased to the instrument's limit of detection at 15.8 m (0.68 mg L^−1^), thus the 15 m sample is considered to be suboxic. We sampled the anoxic hypolimnion at 25 and 31 m, sulfide was present at 31 m.

### Community profiling by 16S rRNA analysis

We obtained between 4,137 and 16,913 high quality 16S rRNA gene reads from each pyrosequenced library from each depth (Table 1). Chloroplast sequences accounted for 5–57% of the reads (Table 1) and made up an increasing proportion of the reads as depth increased. Chloroplast sequences were dominated (>99%) by one OTU, which was ≥99% similar to the 16S rRNA gene sequence from *Picocystis salinarum* CCMP1897 chloroplasts and identical to a plastid sequence retrieved from Mono Lake by Humayoun et al. (2003).

**Table 1:** Summary statistics for 16S rRNA gene pyrosequencing of Mono Lake samples.

**Read statistics**	**Depth (m)**				
	**10**	**15**	**18**	**25**	**31**
Reads passing QC	16,913	8,433	9,978	8,003	4,137
Final reads^a^	15,485	6,031	6,006	3,560	1,588
Contribution of chloroplasts (%)	5	26	36	51	57
OTUs observed (238 total)	98	170	192	181	125

a *After alignment failures, singletons, chloroplasts, and mitochrondia removed*.

**Figure 2:**
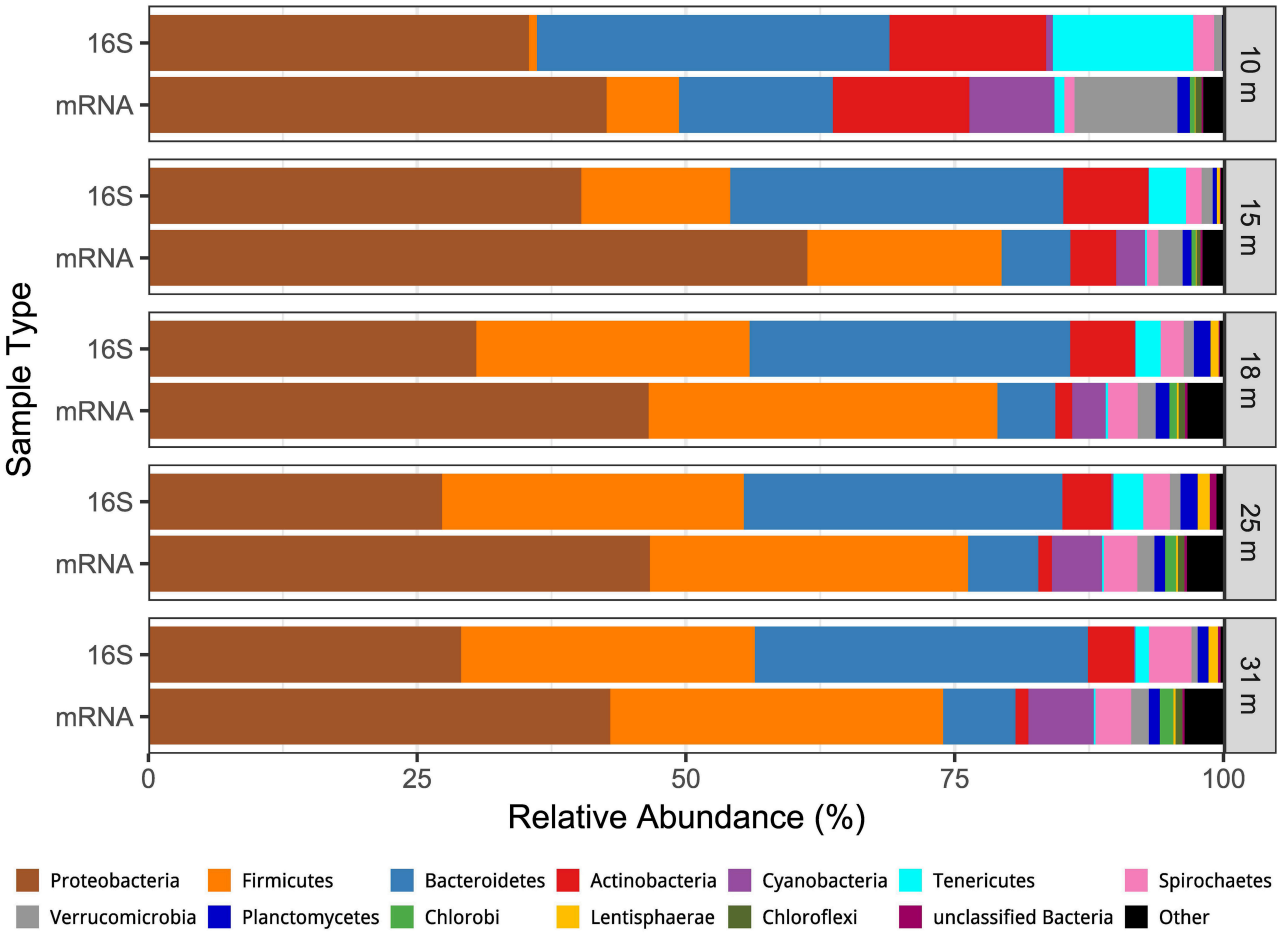
Comparison of the depth distribution of transcriptionally active microbial phyla (mRNA) the depth distribution of phyla derived from 16S rRNA genes.

We obtained a combined total of 238 OTUs (236 Bacteria, 2 Archaea) from all samples and all depths. The distribution by depth of these OTUs, with read counts and relative abundance is presented Table S1, while their full taxonomy is presented in Table S2. Community richness and inverse Simpson diversity were lowest at 10 m, increased at 15–25 m, then decreased at 31 m (Figure S1). Weighted UniFrac analysis of beta diversity (Hamady et al., 2009) showed that the communities were structured by depth, with the 10 and 31 m samples most dissimilar from the others, and the 15, 18, 25, and 31 m samples structured along a gradient with the two MDS axes representing 94.8% of the variation (Figure S1). Proteobacteria (27–40%), Bacteroidetes (30–33%), Firmicutes (1–28%), and Actinobacteria (4–15%) were the most abundant phyla, and only one other phylum (Spirochaetes, 1–4%) made up more than 1% of the population at any depth (Figure 2).

Forty of the 238 observed OTUs were present at all five depths with 34 OTUs greater than 1% relative abundance at any depth, and 10 OTUs greater than 1% relative abundance at all depths (Table 2). These core OTUs made up 60–74% of the overall microbial community at all depths. The most abundant OTU (OTU 8, 5.9–19.9% relative abundance, Table 2) was classified as a Bacteroidetes (class Cytophagia) related to clone ML602J-37, which was retrieved from Mono Lake by Humayoun et al. (2003). Another Cytophagia (represented by clone ML310M-34) was also present at all depths (1.1–7.7%), but was more abundant in samples from below the oxycline. Five additional core OTUs included two Actinobacteria (Microbacteriaceae, 1.8–4.0%; *Nitriliruptor*, 2.0–8.7%), an Alphaproteobacteria (unclassified Rhodobacteraceae, 4.3–6.8%), a Gammaproteobacteria (*Marinicella*, 1.4–5.6%), and *Spirochaeta* (1.3–2.7%).

**Table 2:** OTUs with relative abundance greater than 1% at any depth.

**OTU ID**	**RA (%) by depth (m)**					**Full SILVA taxonomy**
	**10**	**15**	**18**	**25**	**31**	
83	4	3	3	2	2	*Bacteria; Actinobacteria; Actinobacteria; Micrococcales; Microbacteriaceae; unclassified Microbacteriaceae*
3	9	4	3	2	2	*Bacteria; Actinobacteria; Nitriliruptoria; Nitriliruptorales; Nitriliruptoraceae; Nitriliruptor*
162	1	0	0	0	0	*Bacteria; Actinobacteria; Nitriliruptoria; Nitriliruptorales; Nitriliruptoraceae; Nitriliruptor*
22	0	1	2	3	4	*Bacteria; Bacteroidetes; Bacteroidia; Bacteroidales; unclassified Bacteroidales; ML635J-40 aquatic group*
214	0	0	1	1	2	*Bacteria; Bacteroidetes; Bacteroidia; Bacteroidales; unclassified Bacteroidales; ML635J-40 aquatic group*
6	1	5	6	6	8	*Bacteria; Bacteroidetes; Cytophagia; unclassified Cytophagia; Order III Incertae Sedis; ML310M-34*
8	20	14	10	7	6	*Bacteria; Bacteroidetes; Cytophagia; unclassified Cytophagia; Order III Incertae Sedis; ML602J-37*
173	0	1	1	1	1	*Bacteria; Bacteroidetes; Cytophagia; unclassified Cytophagia; Order III Incertae Sedis; ML602J-37*
26	2	2	1	2	1	*Bacteria; Bacteroidetes; Flavobacteria; Flavobacteriales; Cryomorphaceae; Brumimicrobium*
90	1	1	1	1	0	*Bacteria; Bacteroidetes; Flavobacteria; Flavobacteriales; Flavobacteriaceae; Psychroflexus*
9	0	1	1	1	1	*Bacteria; Bacteroidetes; Sphingobacteriia; Sphingobacteriales; Saprospiraceae; uncultured Saprospiraceae*
7	6	3	2	1	1	*Bacteria; Bacteroidetes; unclassified Bacteroidetes; unclassified Bacteroidetes; unclassified Bacteroidetes; ML602M-17*
155	0	0	1	1	1	*Bacteria; Firmicutes; Bacilli; Bacillales; Paenibacillaceae; uncultured Paenibacillaceae*
150	0	6	6	7	7	*Bacteria; Firmicutes; Clostridia; Clostridiales; Ruminococcaceae; unclassified Ruminococcacaeae*
36	0	2	6	5	4	*Bacteria; Firmicutes; Clostridia; Clostridiales; Syntrophomonadaceae; Dethiobacter*
28	0	2	5	6	0	*Bacteria; Firmicutes; Clostridia; Clostridiales; Syntrophomonadaceae; uncultured Syntrophomonadaceae*
194	0	0	0	0	5	*Bacteria; Firmicutes; Clostridia; Clostridiales; Syntrophomonadaceae; uncultured Syntrophomonadaceae*
166	0	0	0	0	2	*Bacteria; Firmicutes; Clostridia; Clostridiales; unclassified Clostridiales; OPB54*
64	0	0	1	1	0	*Bacteria; Planctomycetes; Phycisphaerae; unclassified phycisphaerae; unclassified phycisphaerae; ML-A-10*
81	4	7	5	4	6	*Bacteria; Proteobacteria; Alphaproteobacteria; Rhodobacterales; Rhodobacteraceae; unclassified Rhodobacteraceae*
95	3	1	1	1	0	*Bacteria; Proteobacteria; Betaproteobacteria; Burkholderiales; Alcaligenaceae; GKS98 freshwater group*
20	0	1	2	2	4	*Bacteria; Proteobacteria; Deltaproteobacteria; Desulfobacterales; Desulfobulbaceae; Desulfurivibrio*
80	0	0	0	0	1	*Bacteria; Proteobacteria; Deltaproteobacteria; Desulfovibrionales; Desulfonatronaceae; Desulfonatronum*
12	15	8	5	3	0	*Bacteria; Proteobacteria; Gammaproteobacteria; Chromatiales; Ectothiorhodospiraceae; Spiribacter*
169	0	0	0	0	2	*Bacteria; Proteobacteria; Gammaproteobacteria; Chromatiales; Ectothiorhodospiraceae; Spiribacter*
10	0	3	3	5	6	*Bacteria; Proteobacteria; Gammaproteobacteria; Chromatiales; Ectothiorhodospiraceae; Thioalkalivibrio*
50	1	0	0	0	0	*Bacteria; Proteobacteria; Gammaproteobacteria; Chromatiales; Ectothiorhodospiraceae; Thioalkalivibrio*
13	2	2	1	1	0	*Bacteria; Proteobacteria; Gammaproteobacteria; Oceanospirillales; unclassified Oceanospillales; ML617.5J-3*
41	0	10	7	4	0	*Bacteria; Proteobacteria; Gammaproteobacteria; Thiotrichales; Piscirickettsiaceae; Thiomicrospira*
176	0	0	0	0	2	*Bacteria; Proteobacteria; Gammaproteobacteria; Thiotrichales; Piscirickettsiaceae; Thiomicrospira*
11	6	3	2	1	2	*Bacteria; Proteobacteria; Gammaproteobacteria; unclassified gammaproteobacteria; unclassified gammaproteobacteria; Marinicella*
21	2	1	2	2	3	*Bacteria; Spirochaetes; Spirochaetes; Spirochaetales; Spirochaetaceae; Spirochaeta*
16	13	3	2	1	1	*Bacteria; Tenericutes; Mollicutes; unclassified Mollicutes; unclassified Mollicutes; NB1-n*
158	0	0	0	1	1	*Bacteria; Tenericutes; Mollicutes; unclassified Mollicutes; unclassified Mollicutes; RF9*

*RA%, relative abundance (rounded). Abundances are shaded from low (blue) to high (red)*.

All but three of these OTUs (ML2012 OTUs 80, 150, and 166) were most similar to sequences retrieved from Mono Lake. Additionally, many of the taxonomic assignments were to taxonomic groups represented in the SILVA database by sequences retrieved from Mono Lake (e.g., ML60J37, ML635J-40 aquatic group, ML-A-10; Table 2), suggesting the presence in Mono Lake of organisms representing previously undescribed genera (and in some cases higher taxonomic levels).

### Transcriptionally active taxa

Between 8 and 18 million paired reads were obtained per library, with an average read length of ~240 nt (See Edwardson and Hollibaugh, 2017). At the domain level 68% of the cDNA sequences were affiliated with Bacteria, 30.4% with Eukarya, 0.6% with Archaea, and 0.8% with Viruses. Forty-five to fifty-nine percent of the Bacteria hits to RefSeq proteins were for hypothetical proteins or were misannotations that were subsequently removed from the analysis (see Materials and Methods and Edwardson and Hollibaugh, 2017). Figure 2 compares the phylum-level composition of the transcriptionally active microbial community with the composition determined from analysis of 16S rRNA gene sequences. Sequences from Proteobacteria accounted for 43–61% of the cDNA sequences in libraries from all depths. Firmicutes (7–32% of transcripts) and Bacteroidetes (5–14% of transcripts) were the next most abundant transcriptionally active phyla. Bacteroidetes transcripts were more abundant at 10 m than at other depths, and Firmicutes transcripts were abundant in samples from the anoxic water column (15–31 m). Actinobacteria transcripts were abundant (14%) at 10 m but less abundant at anoxic depths.

**Figure 3:**
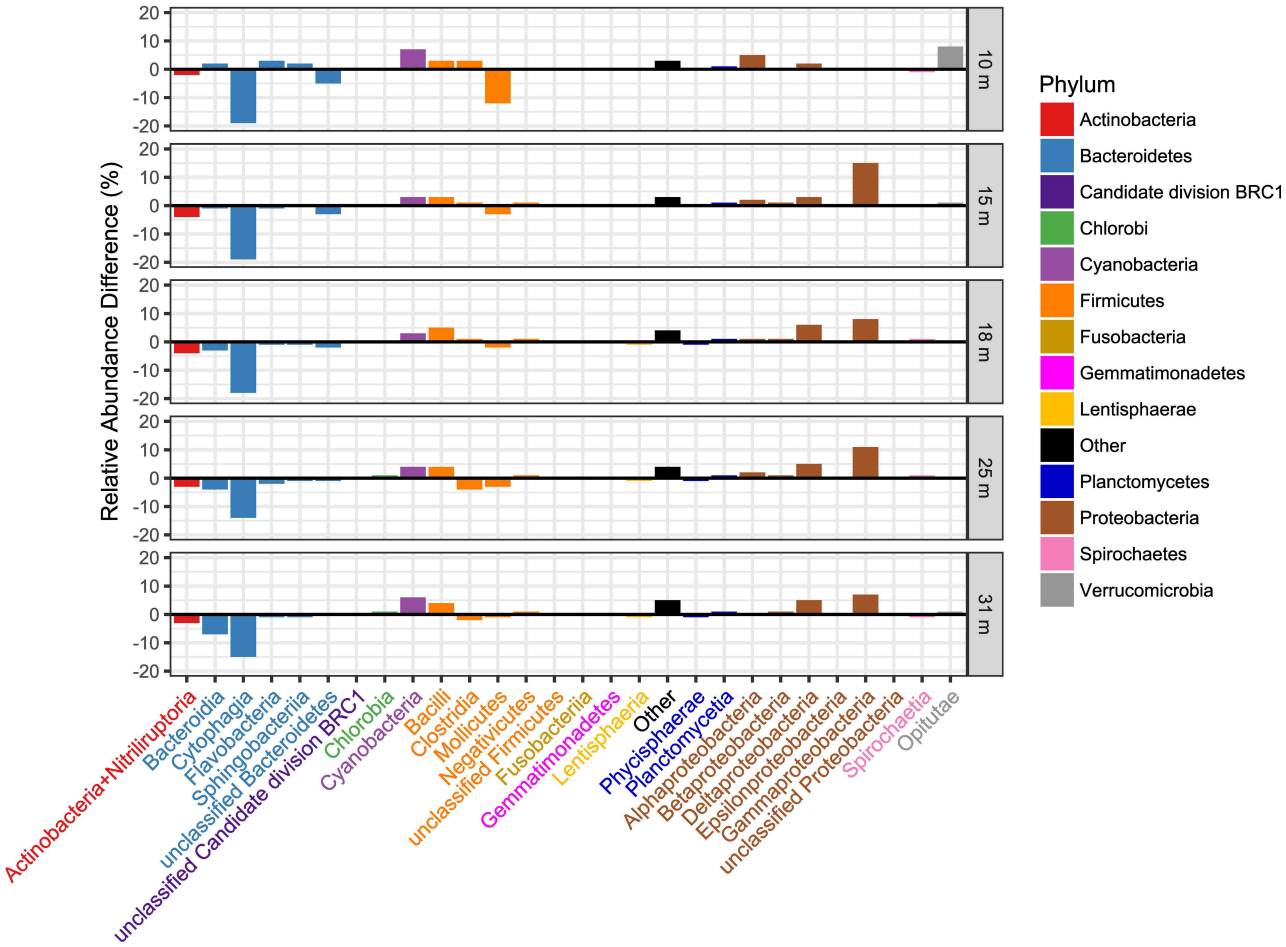
Comparison of the distribution of transcriptionally active taxa with taxa determined from 16S rRNA gene sequences at the taxonomic level of class and by sample depth. The relative abundance of OTUs derived from 16S rRNA was subtracted from the relative abundance of the same OTUs derived from transcript hits, thus positive bars indicate dominance of that class in transcript bins. Class names are colored by phylum as indicated in the legend.

Transcript abundance ranged from ~0.25 to ~1.5 trillion transcripts per liter, on average, at each depth, increasing with depth (Figure S2). Closer examination of the differences in relative abundance derived from 16S gene OTUs vs. transcripts (Figure 3) reveal the greatest differences within the Bacteroidetes and Proteobacteria phyla. In general, the Bacteroidetes are relatively more abundant in the 16S OTU dataset than in the metatranscriptome dataset. The opposite is true for Proteobacteria. The greatest differences at the class level are for the Cytophagia and Bacteroidia classes in the Bacteroidetes (more abundant in 16S OTUs) and in the Delta- and Gammaproteobacteria (more abundant as OTU's derived from transcripts).

We determined the contribution of abundant (>1% of the transcript pool) genera to the metatranscriptome at each depth (Table 3). The genus-level bins that contained the most transcripts in samples from 10 m include *Spiribacter* (4%) and *Thioalkalivibrio* (4%), two Gammaproteobacteria in the Ectothiorhodospiraceae family. Other abundant transcriptionally active taxa from 10 m include the cyanobacteria *Cyanobium* (3%) and *Synechococcus* (2%), as well as Actinobacteria, Bacterioidetes, and Verrucomicrobia. Transcripts from the genus *Thioalkalivibrio*, a haloalkaliphilic Gammaproteobacteria (Sorokin et al., 2001), were abundant at all depths below 15 m. Transcripts from *Thioalkalimicrobium*, another haloalkaliphilic Gammaproteobacteria, were the most abundant bin at 15 m (14% of the libraries). These two taxa accounted for 27% of the transcripts at 15 m. *Clostridium* species were more abundant at depths ≥15 m than at shallower depths, and we observed an increase in the abundance of transcripts from the genus *Dethiobacter*, a sulfide-oxidizing denitrifier in the order Clostridiales (Sorokin et al., 2008; Thorup et al., 2017), at 18 m. At this depth, transcripts assigned to *Dethiobacter* comprised the third largest taxonomic bin following bins for the chemolithotrophic sulfur oxidizers *Thioalkalivibrio* and *Thioalkalimicrobium*, both 9%. We also saw an increase from 18 to 31 m in the abundance of transcripts from known sulfate-reducing genera of the Deltaproteobacteria. Transcripts related to obligate anaerobic Firmicutes and Spirochetes taxa increased in abundance at 25 and 31 m, and a decrease in the abundance of transcripts related to sulfur oxidizing bacteria was seen at these depths as well.

**Table 3:** Genera >1% relative abundance (rounded) in metatranscriptomes at any depth with classification and top genome bins.

**Genus**	**m (%)**					**Phylum**	**Class; Order; Family**	**Dominant Bin (or # of bins)**
	**10**	**15**	**18**	**25**	**31**			
*Ilumatobacter*	1	0	0	0	0	Actinobacteria	Actinobacteria; Acidimicrobiales; Acidimicrobiaceae	*Ilumatobacter coccineum* YM16-304
*Leifsonia*	1	0	0	0	0	Actinobacteria	Actinobacteria; Actinomycetales; Microbacteriaceae	5
*Streptomyces*	1	0	0	0	0	Actinobacteria	Actinobacteria; Actinomycetales; Streptomycetaceae	>10
*Anaerophaga*	0	0	0	0	1	Bacteroidetes	Bacteroidia; Bacteroidales; Marinilabiliaceae	*Anaerophaga thermohalophila Anaerophaga* sp. HS1
*Fluviicola*	1	0	0	0	0	Bacteroidetes	Flavobacteriia; Flavobacteriales; Cryomorphaceae	*Fluviicola taffensis* DSM 16823
*Owenweeksia*	1	0	0	0	0	Bacteroidetes	Flavobacteriia; Flavobacteriales; Cryomorphaceae	*Owenweeksia hongkongensis* DSM 17368
*Flavobacterium*	1	0	0	0	0	Bacteroidetes	Flavobacteriia; Flavobacteriales; Flavobacteriaceae	>10
*Psychroflexus*	1	0	0	0	0	Bacteroidetes	Flavobacteriia; Flavobacteriales; Flavobacteriaceae	*Psychroflexus gondawanenis Psychroflexus torquis Psychroflexus tropicus*
*Pelodictyon*	0	0	0	0	1	Chlorobi	Chlorobia; Chlorobiales; Chlorobiaceae	*Chlorobium luteolum* DSM 273 *Pelodictyon phaeoclathratiforme* BU-1
*Cyanobium*	3	0	0	0	0	Cyanobacteria	unclassified; Chroococcales; unclassified	*Cyanobium* sp. PCC 7001 Cyanobium gracile PCC 6307
*Synechococcus*	2	0	0	0	0	Cyanobacteria	unclassified; Chroococcales; unclassified	>10
*Trichodesmium*	1	1	1	3	4	Cyanobacteria	unclassified; Oscillatoriales; unclassified	*Trichodesmium erythraeum* IMS101
*Bacillus*	1	1	2	1	1	Firmicutes	Bacilli; Bacillales; Bacillaceae	>10 including *Bacillus thuringiensis Bacillus selenitireducens* MLS10 *Bacillus smithii Bacillus cellulosilyticus*
*Paenibacillus*	0	0	1	1	1	Firmicutes	Bacilli; Bacillales; Paenibacillaceae	>10
*Staphylococcus*	0	0	0	0	1	Firmicutes	Bacilli; Bacillales; Staphylococcaceae	*Staphylococcus hominis* and others
*Enterococcus*	1	1	1	0	0	Firmicutes	Bacilli; Lactobacillales; Enterococcaceae	Enterococcus faecalis *Enterococcus faecium* and others
*Alkaliphilus*	0	0	1	1	1	Firmicutes	Clostridia; Clostridiales; Clostridiaceae	*Alkaliphilus metalliredigens* QYMF *Alkaliphilus oremlandii* OhILAs
*Clostridium*	1	4	5	4	4	Firmicutes	Clostridia; Clostridiales; Clostridiaceae	*Clostridium difficile Clostridium thermocellum Clostridium clariflavum Clostridium termitidis Clostridium papyrosolvens*
*Desulfosporosinus*	0	0	1	1	1	Firmicutes	Clostridia; Clostridiales; Peptococcaceae	*Desulfosporosinus orientis* DSM 765 and 4 others
*Desulfotomaculum*	0	1	2	2	2	Firmicutes	Clostridia; Clostridiales; Peptococcaceae	7
*Acetivibrio*	0	1	0	0	0	Firmicutes	Clostridia; Clostridiales; Ruminococcaceae	*Acetivibrio cellulolyticus*
*Dethiobacter*	0	2	5	5	5	Firmicutes	Clostridia; Clostridiales; Syntrophomonadaceae	*Dethiobacter alkaliphilus*
*Halanaerobium*	0	0	1	1	1	Firmicutes	Clostridia; Halanaerobiales; Halanaerobiaceae	*Halanaerobium hydrogeniformans Halanaerobium saccharolyticum Halanaerobium prevalens* DSM 2228
*Halothermothrix*	0	0	1	1	1	Firmicutes	Clostridia; Halanaerobiales; Halanaerobiaceae	*Halothermothrix orenii* H168
*Acetohalobium*	0	0	1	1	1	Firmicutes	Clostridia; Halanaerobiales; Halobacteroidaceae	*Acetohalobium arabaticum* DSM 5501
*Natranaerobius*	0	0	1	1	1	Firmicutes	Clostridia; Natranaerobiales; Natranaerobiaceae	*Natranaerobius thermophilus*
*Rhodopirellula*	1	0	0	0	0	Planctomycetes	Planctomycetia; Planctomycetales; Planctomycetaceae	6
*Paracoccus*	0	1	0	0	0	Proteobacteria	Alphaproteobacteria; Rhodobacterales; Rhodobacteraceae	5
*Rhodobacter*	1	1	1	1	1	Proteobacteria	Alphaproteobacteria; Rhodobacterales; Rhodobacteraceae	*Rhodobacter sphaeroides Rhodobacter capsulatus Rhodobacter* sp. CACIA14H1
*Roseobacter*	1	1	0	0	0	Proteobacteria	Alphaproteobacteria; Rhodobacterales; Rhodobacteraceae	Roseobacter sp. AzwK-3b and 6 other species
*Roseovarius*	1	1	0	0	0	Proteobacteria	Alphaproteobacteria; Rhodobacterales; Rhodobacteraceae	*Roseovarius* sp. 217 *Roseovarius* sp. TM1035 *Roseovarius nubinhibens*
*Ruegeria*	0	1	0	0	0	Proteobacteria	Alphaproteobacteria; Rhodobacterales; Rhodobacteraceae	7
*Desulfatibacillum*	0	0	0	1	1	Proteobacteria	Deltaproteobacteria; Desulfobacterales; Desulfobacteraceae	*Desulfatibacillum alkenivorans* AK-01
*Desulfococcus*	0	0	0	1	2	Proteobacteria	Deltaproteobacteria; Desulfobacterales; Desulfobacteraceae	*Desulfatibacillum multivorans Desulfatibacillum oleovorans* Hxd3
*Desulfurivibrio*	0	1	2	0	0	Proteobacteria	Deltaproteobacteria; Desulfobacterales; Desulfobulbaceae	*Desulfurivibrio alkaliphilus* AHT2
*Desulfonatronospira*	0	0	0	1	1	Proteobacteria	Deltaproteobacteria; Desulfovibrionales; Desulfohalobiaceae	*Desulfonatronospira thiodismutans*
*Desulfovibrio*	0	0	1	1	2	Proteobacteria	Deltaproteobacteria; Desulfovibrionales; Desulfovibrionaceae	>10
*Geobacter*	0	0	1	0	1	Proteobacteria	Deltaproteobacteria; Desulfuromonadales; Geobacteraceae	8
*Delta proteobacterium*	0	1	2	1	1	Proteobacteria	Deltaproteobacteria; unclassified Deltaproteobacteria; unclassified Deltaproteobacteria	*deltaproteobacterium* MLMS-1 *deltaproteobacterium* NaphS2
*Marinobacter*	1	0	0	0	0	Proteobacteria	Gammaproteobacteria; Alteromonadales; Alteromonadaceae	10
*Alkalilimnicola*	1	0	0	0	0	Proteobacteria	Gammaproteobacteria; Chromatiales; Ectothiorhodospiraceae	*Alkalilimnicola ehrlichii* MLHE-1
*Spiribacter*	4	1	0	0	0	Proteobacteria	Gammaproteobacteria; Chromatiales; Ectothiorhodospiraceae	*Spiribacter salinus* M19-40
*Thioalkalivibrio*	4	13	9	12	9	Proteobacteria	Gammaproteobacteria; Chromatiales; Ectothiorhodospiraceae	*Thioalkalivibrio nitratireducens Thioalkalivibrio sulfidophilus* HL-EbGr7 *Thioalkalivibrio* sp. K90mix *Thioalkalivibrio thiocyanodenitrificans* and others
*Escherichia*	2	2	2	1	1	Proteobacteria	Gammaproteobacteria; Enterobacteriales; Enterobacteriaceae	*Escherichia coli* strains
*Methylomicrobium*	0	1	0	0	0	Proteobacteria	Gammaproteobacteria; Methylococcales; Methylococcaceae	*Methylomicrobium alcaliphilum* 20Z *Methylomicrobium buryatense Methylomicrobium album*
*Halomonas*	2	1	0	0	0	Proteobacteria	Gammaproteobacteria; Oceanospirillales; Halomonadaceae	14
*Pseudomonas*	1	1	0	0	0	Proteobacteria	Gammaproteobacteria; Pseudomonadales; Pseudomonadaceae	>10
*Thioalkalimicrobium*	0	14	9	6	5	Proteobacteria	Gammaproteobacteria; Thiotrichales; Piscirickettsiaceae	*Thioalkalimicrobium cyclicum* ALM1
*Thiomicrospira*	0	4	3	2	1	Proteobacteria	Gammaproteobacteria; Thiotrichales; Piscirickettsiaceae	*Thiomicrospira crunogena* XCL-2 *Thiomicrospira arctica Thiomicrospira halophila*
*Gamma proteobacterium*	1	0	0	0	0	Proteobacteria	Gammaproteobacteria; unclassified gammaproteobacteria; unclassified gammaproteobacteria	9
*Vibrio*	1	1	0	0	0	Proteobacteria	Gammaproteobacteria; Vibrionales; Vibrionaceae	*Vibrio parahaemolyticus* and others
*Sphaerochaeta*	0	0	1	1	1	Spirochaetes	Spirochaetia; Spirochaetales; Spirochaetaceae	*Sphaerochaeta pleomorpha* str. Grapes
*Spirochaeta*	1	0	1	1	2	Spirochaetes	Spirochaetia; Spirochaetales; Spirochaetaceae	*Spirochaeta africana* DSM 8902 *Spirochaeta alkalica Spirochaeta smaragdinae* DSM 11293
*Opitutaceae bacterium*	2	0	0	0	0	Verrucomicrobia	Opitutae; Opitutales; Opitutaceae	*Opitutaceae* bacterium TAV1
*Opitutus*	2	0	0	0	0	Verrucomicrobia	Opitutae; Opitutales; Opitutaceae	*Opitutus terrae* PB90-1
*Coraliomargarita*	2	1	0	0	0	Verrucomicrobia	Opitutae; Puniceicoccales; Puniceicoccaceae	*Coraliomargarita akajimensis* DSM 45221
*Verrucomicrobiae bacterium*	2	0	0	0	0	Verrucomicrobia	Verrucomicrobiae; Verrucomicrobiales; unclassified Verrucomicrobiales	*Verrucomicrobiae* bacterium DG1235

*Abundances are shaded from low (blue) to high (red). Phylum colors correspond to the legend in Figure 2*.

### Phylogenetic analysis

**Figure 4:**
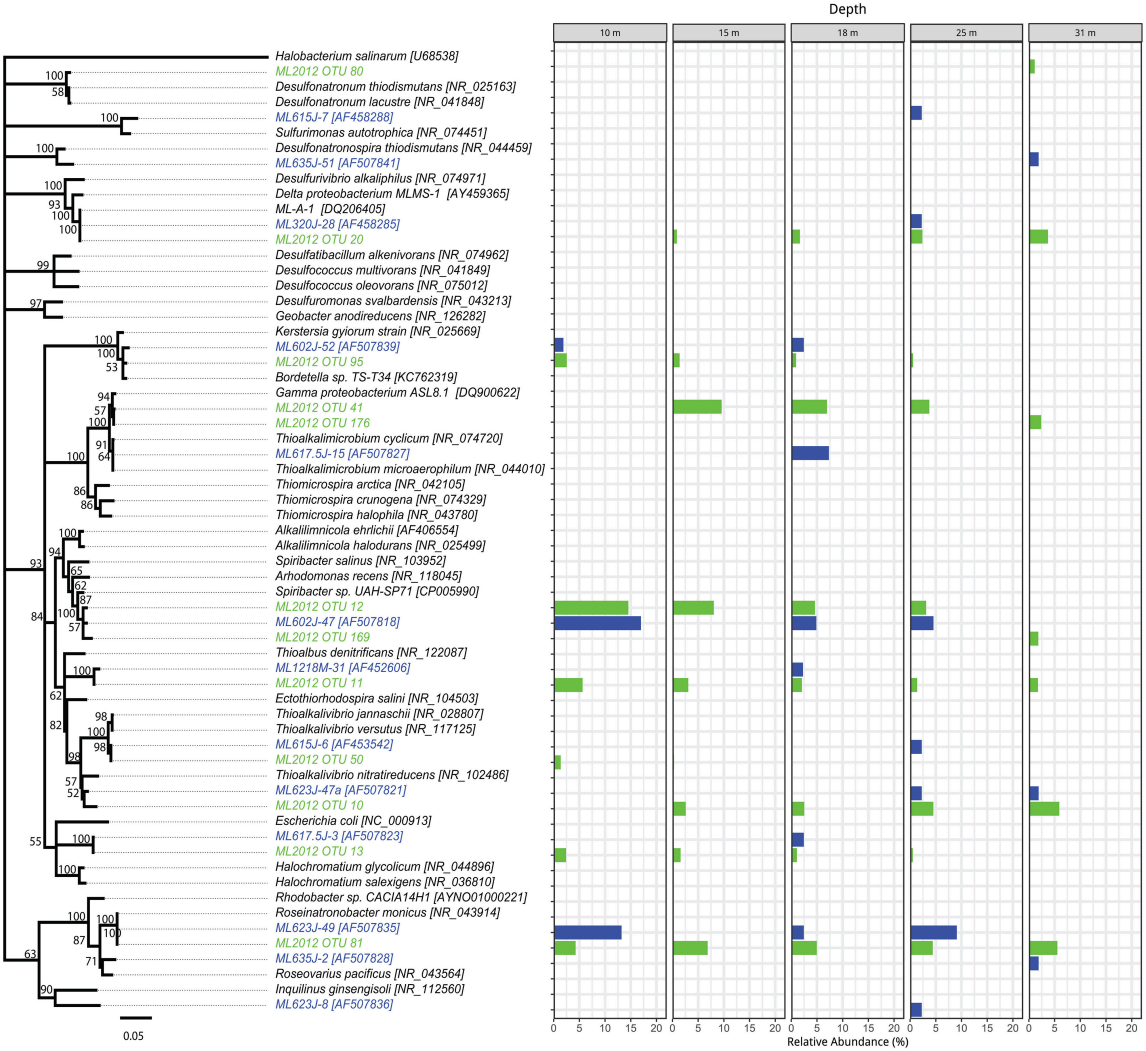
Phylogenetic tree showing the relative abundance of Proteobacteria OTUs by depth. Taxa and bars shown in green represent 16S rRNA OTUs derived from tag pyrosequencing. Taxa and bars shown in blue represent 16S rRNA OTUs derived from sequences of cloned amplicons reported in Humayoun et al. (2003). Taxa shown in black represent 16S rRNA gene reference sequences for bins accounting for >1% relative abundance in the metatranscriptome from that depth (both samples combined). The outgroup is *Halobacterium salinarum*.

**Figure 5:**
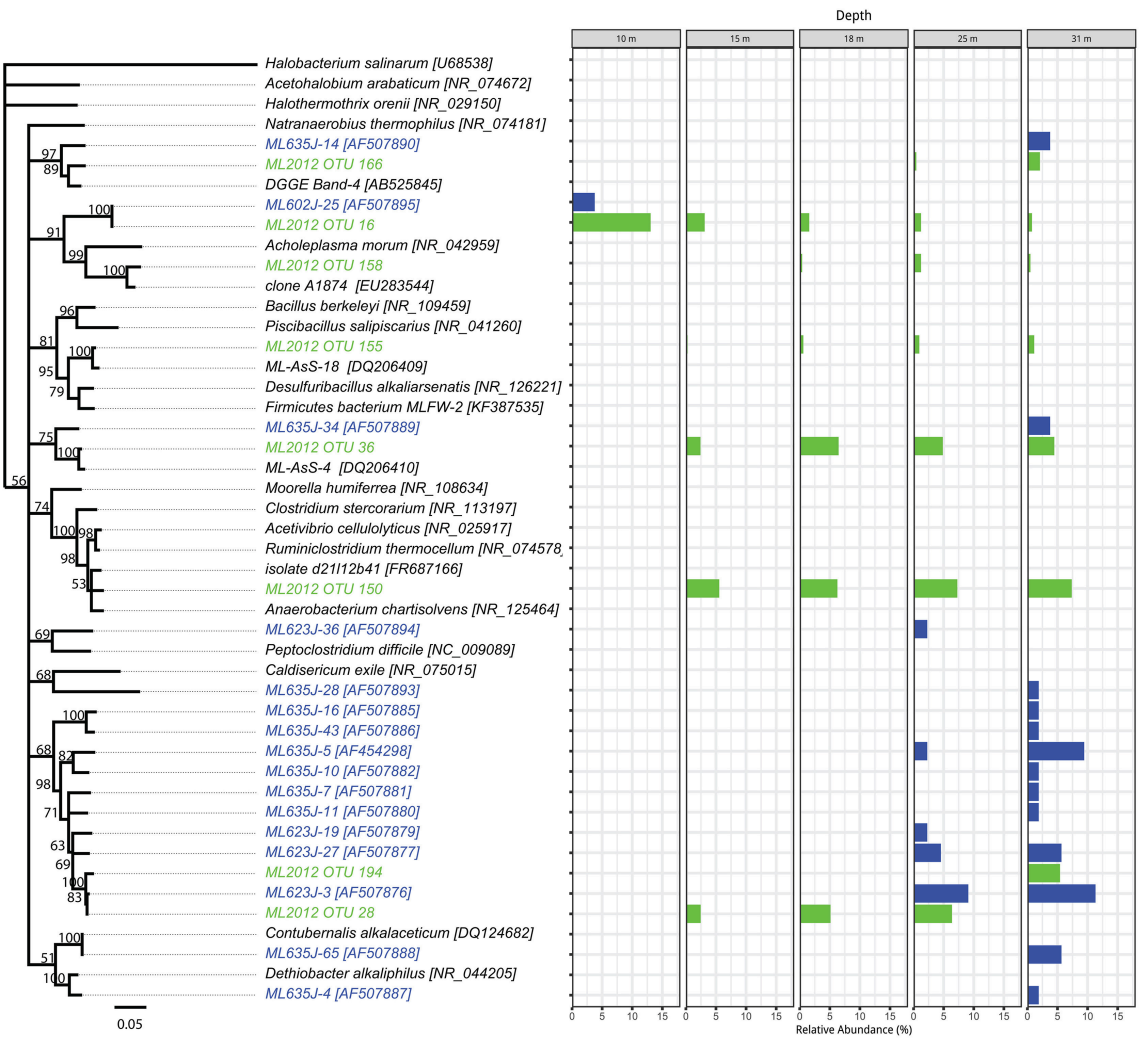
Phylogenetic tree showing the relative abundance of Firmicutes OTUs by depth. Taxa and bars shown in green represent 16S rRNA OTUs derived from tag pyrosequencing. Taxa and bars shown in blue represent 16S rRNA OTUs derived from sequences of cloned amplicons reported in Humayoun et al. (2003). Taxa shown in black represent 16S rRNA reference sequences for bins accounting for >1% relative abundance in the metatranscriptome from that depth (both samples combined). The outgroup is *Halobacterium salinarum*.

**Figure 6:**
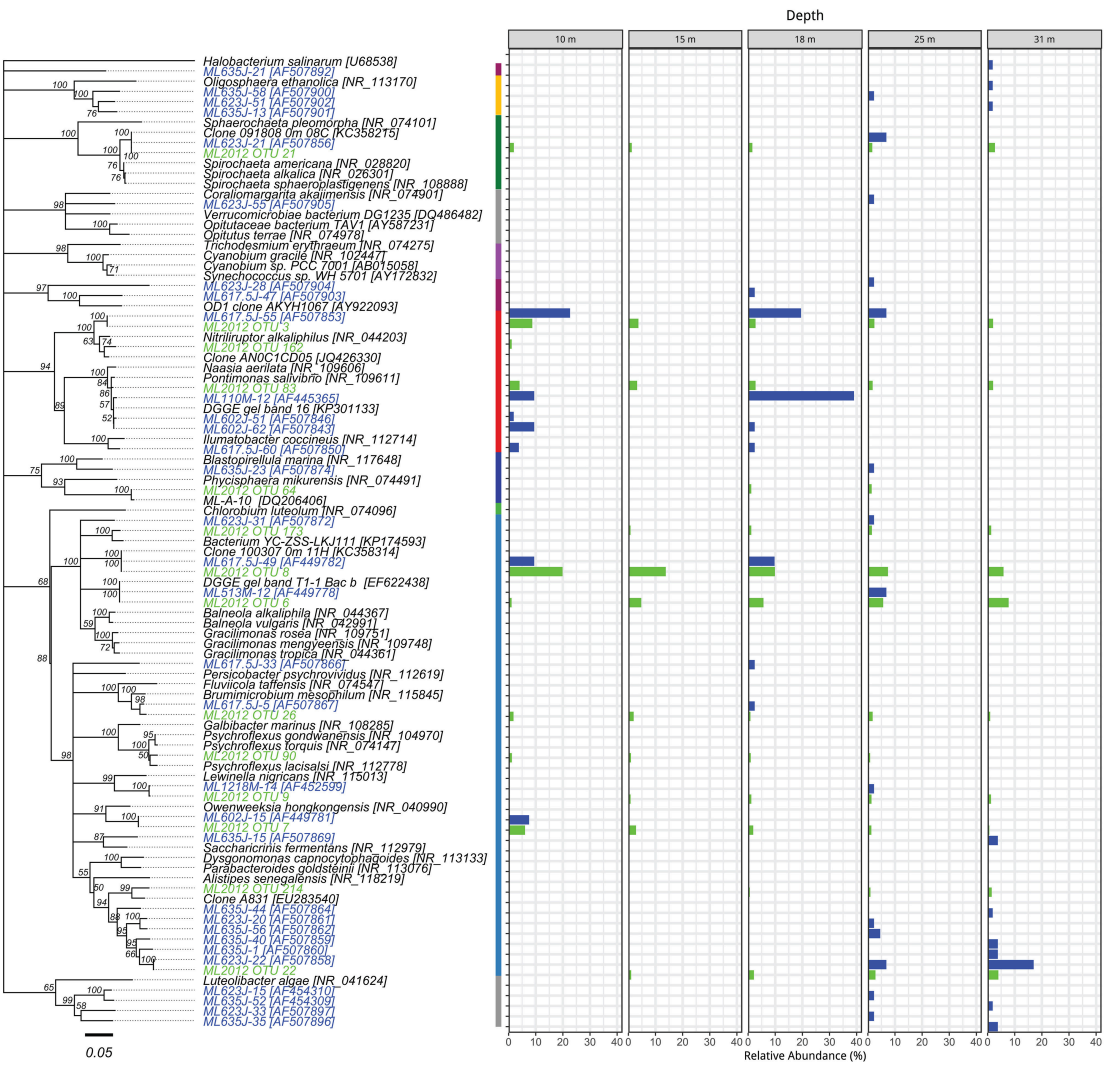
Phylogenetic tree showing the relative abundance of OTUs from Actinobacteria, Bacteroidetes, and other bacteria by depth. The colored segments of the vertical bar between the tree and the charts indicates the phylum associated with the adjacent branch of the tree, colors correspond to the legend of Figure 2. Taxa and bars shown in green represent 16S rRNA OTUs derived from tag pyrosequencing. Taxa and bars shown in blue represent 16S rRNA OTUs derived from sequences of cloned amplicons reported in Humayoun et al. (2003). Taxa shown in black represent 16S rRNA gene reference sequences for bins accounting for >1% relative abundance in the metatranscriptome from that depth (both samples combined). The outgroup is *Halobacterium salinarum*.

The top BLASTN hits to the NCBI nr database of representative sequences from pyrosequenced OTUs were used as reference sequences in phylogenetic trees (Figures 4–6). Eleven OTUs from pyrosequenced libraries were identical to OTUs from the Humayoun et al. (2003) Sanger-sequenced libraries. The relative abundances of OTUs in the Humayoun et al. (2003) libraries, the pyrosequenced libraries, and OTUs derived from taxonomic assignments of metatranscriptomic sequences were similar; however, there were large discrepancies in a few cases (Figure 3). The relative abundance of Actinobacteria OTU 83 was only 3% in pyrosequenced libraries from 18 m, whereas the most closely related OTU in the 17.5 m sample from the Humayoun et al. (2003) dataset accounted for almost 40% of all sequences in our 18 m sample. In addition, our pyrosequenced libraries contained no OTUs corresponding to a group of Firmicutes OTUs from 23 and 35 m in Humayoun et al. (2003). Sequences from this group were not closely related to any 16S rRNA gene reference sequence, with the closest hit being to *Dethiobacter alkaliphilus* at ~90% identity. In fact, the abundant OTUs within the Firmicutes (and Tenericutes, which group phylogenetically within the Firmicutes) from pyrosequenced libraries were only 87–96% similar to 16S reference sequences currently in the NCBI database. In contrast, all of the Proteobacteria OTUs from our pyrosequenced libraries (Figure 4), had ≥97% identity to a sequence in the NCBI database. Some of the OTUs (*Dethiobacter* OTU 36, Paenibacillaceae OTUs 155 and 64, *Desulfurivibrio* OTU 20) that we obtained were most closely related to sequences obtained from sulfide-oxidizing, arsenate-reducing enrichment cultures raised from Mono Lake water (Hollibaugh et al., 2006), rather than to sequences retrieved directly from a water column sample. The other major OTUs in the pyrosequence database consisted of Verrucomicrobia, Actinobacteria, Bacteroidetes (Flavobacteria, Chitinophaga), Spirochetes, Cyanobacteria, and Planctomycetes (Figure 6). None of the OTUs we obtained by pyrosequencing were related to the *Cyanobacteria* or *Chlorobium* OTUs present in metatranscriptome taxonomic bins. This could be due to specificities of the primers we used to amplify 16S rRNA genes.

## Discussion

Unlike a previous study of the phylogenetic composition of Mono Lake microbial communities (Humayoun et al., 2003), we found a set of OTUs that were present at all depths in the lake, possibly as a result of sampling at the beginning, vs. the end, of a period of meromixis, although the low sequencing depth of the previous analysis could have led to missing these taxa. The relative abundance of these OTUs did not vary greatly between depths. In some cases, their relative abundance was greater than their relative abundance in metatranscriptomic bins. This could indicate presence of inactive or dormant cells (e.g., spores) or relic DNA (Carini et al., 2016). Seven OTUs made up 23–45% of the community at all depths. These include two Cytophagia OTUs (12–21% relative abundance) most closely related (90–93% identity) to *Gracilimonas* (a facultative aerobe) and *Balneola* (aerobic) species (Urios et al., 2008; Choi et al., 2009). An Alphaproteobacteria (Rhodobacteraceae) OTU (4–7% relative abundance) was 99% similar to purple non-sulfur *Roseinatronobacter monicus* isolated from Mono Lake (Boldareva et al., 2007). This species is an obligate aerobe. Related *Rhodobaca* species can grow under anaerobic conditions if illuminated, thus the persistence of these OTUs just below the oxycline might be a consequence of photoheterotrophic “maintenance” or, alternatively, the organism might be associated with microaerophilic conditions maintained by oxygenic photosynthesis of *Picocystis*, which is abundant at this depth. However, it is unlikely that irradiance is adequate to support photoheterotrophy or oxygenic photosynthesis below the chemocline (Figure 1). Recovery of this OTU from those depths may be due to the presence of DNA from cells in a stationary phase, or it is a relic (Carini et al., 2016) left over from the last deep mixing event, or to vertical transport of cells associated with sinking particles.

An OTU related to *Nitriliruptor* species (Actinobacteria) contributed 4–7% of the population at all depths. The most closely related strain is the aerobe *Nitriliruptor alkaliphilus*, an isobutyronitrile-degrading haloalkaliphile isolated from enrichment cultures inoculated with water from Soda Lakes (Sorokin et al., 2009). The genome of *Nitriliruptor alkaliphilus* was released after we analyzed our metatranscriptomes; however, if this genome had been included in the analysis it is likely that it would have recruited hits. This observation serves to underline the taxonomic biases of the approaches we tested (metagenomics/metatranscriptomics vs. amplicon sequencing) and sequence databases (a broader 16S rRNA reference database vs. a narrower reference database of sequenced genomes).

*Spiribacter* has been found previously in moderately halophilic environments and is a strict aerobe, which could indicate why it was only found at oxic (10 m) and suboxic (15 m) depths in Mono Lake. *Thioalkalivibrio* has been isolated previously from Soda Lakes, including Mono Lake (Sorokin et al., 2001, 2002). A *Cyanobium* strain was isolated from Mono Lake samples and characterized, but counts by epifluorescence microscopy showed that cells were more abundant in samples from aphotic depths than nearer the surface (Budinoff and Hollibaugh, 2007). However, we found more transcripts of this organism at 10 m than at depth, supporting the hypothesis that the *Cyanobium* cells found in the aphotic zone are inactive or represent a flux sinking from the euphotic zone of the lake.

Soda lakes typically contain an abundance of diverse reduced sulfur compounds and one of the major processes in soda lake biogeochemistry is sulfur cycling (Sorokin, 2011). Sulfur cycling microbial taxa from soda lakes are well-represented by cultured isolates (Sorokin et al., 2013). As expected, OTUs representing many sulfur-oxidizing bacteria were found in both the pyrosequenced 16S rRNA gene libraries and in taxonomic bins generated from metatranscriptomic samples collected at 15–31 m.

*Thioalkalimicrobium* (OTU 41) and *Thioalkalivibrio* (OTUs 10 and 50) made up 5–12% of the OTUs in pyrosequenced libraries and 12–24% of the OTUs derived from metatranscriptomic taxonomic bins in these samples. The abundance of *Thioalkalimicrobium* decreased with depth but *Thioalkalivibrio* abundance increased in anoxic water below 15 m. Both taxa should have decreased in abundance with depth, as they are both believed to be aerobic sulfur oxidizers (Sorokin et al., 2002). Although some *Thioalkalivibrio* have the ability to use alternative terminal electron acceptors such as nitrate (Sorokin, 2011), we did not find any evidence that this process was occurring (e.g., transcripts of nitrate reductase) in the metatranscriptomes (data not shown). The likely role of these two organisms as the dominant sulfur-oxidizing bacteria in Mono Lake was not surprising, as both *Thioalkalivibrio jannaschii* and *Thioalkalimicrobium cyclicum* were isolated from Mono Lake (Sorokin et al., 2002). In addition, *Dethiobacter alkaliphilus*, the most abundant metatranscriptomic taxonomic bin in the Firmicutes phylum, is a sulfide-oxidizing denitrifier (Sorokin et al., 2008; Thorup et al., 2017).

Many different sulfate-reducing bacteria (various Deltaproteobacteria and OTU 20) appear between 18 and 31 m. This was expected as sulfate reduction rates are higher at depth in the lake, particularly when it is strongly stratified (Oremland et al., 2000).

Previous work on nitrogen cycling in Mono Lake (Carini and Joye, 2008) identified a peak in ammonia oxidation rates at 12–14 m, coinciding with the presence of ammonia-oxidizing bacteria of the genus *Nitrosomonas*. We found few (<1% relative abundance) OTUs related to ammonia-oxidizing Bacteria or Archaea in either our pyrosequenced libraries or in taxonomic bins derived from metatranscriptomes. Moreover, metatranscriptomes contained little evidence for the presence of ammonia-oxidizing Archaea. Transcripts of genes from ammonia-oxidizing bacteria such as *Nitrosomonas*, which was identified as the dominant taxon of ammonia-oxidizing bacteria by Carini and Joye (2008), were in low abundance, and we found no OTUs representing other known nitrifying bacteria in the pyrosequenced libraries. One possible explanation for the activity Carini and Joye (2008) observed is the presence in Mono Lake of methanotrophs (e.g., *Methylomicrobium* and *Methyloglobus*, Nercessian et al., 2005), which were also present in the metatranscriptomes. Some methane-oxidizers can also oxidize ammonia (Nyerges and Stein, 2009).

We observed increasing relative abundance with depth (1–4%) of transcripts that were identified initially as the cyanobacteria *Trichodesmium*. We assembled these transcripts using the Geneious assembler with default settings to analyze them further. The consensus sequences obtained were searched against contigs from the Mono Lake *Picocystis* draft genome (C. Saltikov, unpublished) using BLASTN. Approximately 90% of the hits were ≥97% identical and 25% (278/1,180) were 100% identical to *Picocystis*. Further, these hits were to a single *Picocystis* consensus sequence of 87,248 bp. This sequence is 86% similar (nucleotide identity) to the *Picocystis salinarum* chloroplast from San Francisco Bay (Lemieux et al., 2014), indicating that the contig was likely derived from *Picocystis* chloroplasts. Interestingly, a *Trichodesmium*-related Oscillatoriales species, *Phormidium*, which was identified in nitrogen-fixing aggregates in Mono Lake (Oremland, 1990), was not detected in our samples, likely because it grows attached to solid substrates at the lake's edge and floats at the lake's surface when detached.

### Diversity relative to previous studies and other soda lakes

Mono Lake microbial communities seem to have remained stable, or returned to the same composition, after multiple mixing events over the 12 years between sampling efforts, despite the significant physicochemical differences in the lake following prolonged meromixis when sampled in 2000 vs. 2012. A number of OTUs that were abundant in the Humayoun et al. (2003) study were not represented by any OTUs in our pyrosequencing dataset. Some of the differences between these studies can be attributed to sequencing depth and primer biases, but Humayoun et al. (2003) determined the composition of the Mono Lake microbial community after 5 years of stratification. In contrast, our sampling effort occurred after only ~18 months of stratification (only 1 winter of partial mixing). The major ion composition and pH of Mono Lake are stable (Domagalski et al., 1989), thus the main physiochemical differences in the lake between July 2000 when Humayoun et al. (2003) sampled and July 2012 when we sampled was higher hypolimnion concentrations of reduced species like sulfide (>2,500 vs. 30 μM) and ammonia (>400 μM in 2000), that are produced by microbial activity or that diffused into the bottom water from sediments. The high sulfide concentration results in the formation of thioarsenic compounds in the lake (Hollibaugh et al., 2005; Planer-Friedrich et al., 2007) so that most of the reduced As in the bottom water of the lake (~200 μM) was present in the form of higher order thioarsenic species in 2000 (Hollibaugh et al., 2005), whereas most of the reduced As was present as arsenite when we sampled in 2012 (Figure 1). Furthermore, at the elevated sulfide concentrations encountered in 2000, the reduced S pool is likely to contain species like polysulfides (Domagalski et al., 1990). Thus, differences in the composition of the microbial community between 2000 and 2012, particularly at depths below the chemocline, may reflect selection for taxa that are adapted to the higher sulfide and more strongly reducing conditions encountered in 2000. The microbial community encountered in the present study may represent a more diverse, and less stratified, set of taxa as a result of the rapidly changing conditions following the recent (6–8 months) establishment of meromixis.

The distribution of alpha diversity we found was similar to that reported by Humayoun et al. (2003), where community diversity increased with depth. However, we found a decrease in community diversity at 31 m. Similar changes in community diversity with depth were found in Lake Kivu, Africa, with greater diversity reported in the anoxic regions of the lake (Inceoglu et al., 2015). The microbial communities found in Soap Lake (Washington) also have a composition similar to Mono Lake and to other soda lakes, with greater diversity in the deep, sulfidic region of the water column (Dimitriu et al., 2008). Several other studies (e.g., Wani et al., 2006; Lanzén et al., 2013) found that the composition of microbial communities in other Soda Lake environments is similar to those we found in Mono Lake. 16S rRNA gene sequences most similar to Mono Lake sequences ML1228J-1 (Firmicutes) and ML635J-40 (Bacteroidetes) were recovered from organisms cultivated from the interior of ikaite columns (pH > 10) found in the Ikka Fjord in Greenland (Schmidt et al., 2006). The physiological requirements for survival under the conditions encountered in these environments likely limits the diversity of organisms that can survive there (Oren, 1999; Mesbah and Wiegel, 2012).

## Author contributions

JH and CE designed the study, JH and CE collected the samples, CE analyzed the samples and performed bioinformatics analysis, CE and JH wrote the paper.

### Conflict of interest statement

The authors declare that the research was conducted in the absence of any commercial or financial relationships that could be construed as a potential conflict of interest.
